# A Twist on Adolescent Abdominal Pain in the Emergency Department

**DOI:** 10.7759/cureus.27371

**Published:** 2022-07-27

**Authors:** Trevor Lofgran, Ron Koury

**Affiliations:** 1 Emergency Medicine, Orange Park Medical Center, Orange Park, USA

**Keywords:** pediatrics emergency, emergency medicine, adolescent, abdominal pain, sigmoid volvulus

## Abstract

Abdominal pain is a common complaint in pediatric patients in the emergency department (ED). Evolutions in clinical practice have shifted away from computed tomography (CT) to ultrasound (US) in assessing abdominal pain. However, ultrasound may not reliably rule out critical diagnoses. We present a 15-year-old male with intermittent suprapubic abdominal pain. Subsequent CT imaging showed swirling mesenteric vessels with a dilated sigmoid colon. In adolescent abdominal pain, sigmoid volvulus (SV), although rare, should be considered. Clinicians should avoid anchoring bias by maintaining a broad differential. Definitive care is surgical with resection to prevent recurrence.

## Introduction

Acute abdominal pain is a common presenting symptom of pediatric patients in the emergency department (ED). While the differential is often broad, the diagnosis can be narrowed depending on patient age and gender, with the most common surgical emergency in adolescents being appendicitis [1]. Adolescents with concerning histories and/or physical examination findings require additional laboratory testing and diagnostic imaging. Evolving practice patterns have shifted away from computed tomography (CT) scans in pediatric and adolescent populations to the use of ultrasound (US) in the assessment of abdominal pain.

Sigmoid volvulus (SV) is a very rare cause of acute abdominal pain that is often a missed or delayed diagnosis in adolescents [2]. It is caused by the twisting of the mesenteric vessels of the sigmoid colon and more commonly affects males in the seventh and eighth decade of life [3-5]. Prior case reports of adolescents have correlated it with a history of constipation, redundant loops of bowel, Hirschsprung’s disease, or debilitated states [6]. Signs and symptoms are variable with nonspecific pain patterns that mimic other etiologies [2,7]. Onset can be sudden or insidious and is occasionally relieved with bowel movements due to spontaneous intermittent detorsion [7,8]. The classic radiographic findings of “coffee bean” or “inner tube” signs on X-rays are unreliable and should be followed by either a CT scan or surgical evaluation [9]. If left untreated, SV can lead to bowel obstruction, tissue ischemia, and even necrosis [7].

Sigmoid volvulus should be considered a “can’t miss” diagnosis for all cases of pediatric abdominal pain. Complications can be life-threatening, and clinicians should avoid anchoring solely on a single piece of information that would suggest other more common diagnoses such as appendicitis and constipation. Diagnostic evaluation and management should be expanded in atypical presentations. Here, we present a case of sigmoid volvulus in an otherwise healthy adolescent male.

## Case presentation

A Caucasian 15-year-old male presented to the ED with suprapubic abdominal pain that began a few hours prior to arrival. Symptoms progressively worsened until, while at school, he called his parents to bring him to the hospital. The pain was non-radiating, crampy, and intermittent with episodes lasting approximately one minute before spontaneous resolution. A week prior to presentation, the patient had been evaluated at a different facility for an episode of sudden-onset suprapubic pain that was followed by a sudden urge to defecate. He had a brief syncopal episode shortly after the onset of symptoms, followed by subsequent resolution of his pain. He denied prior medical and surgical history and complaints of fevers, chills, nausea, or constipation.

**Figure 1:**
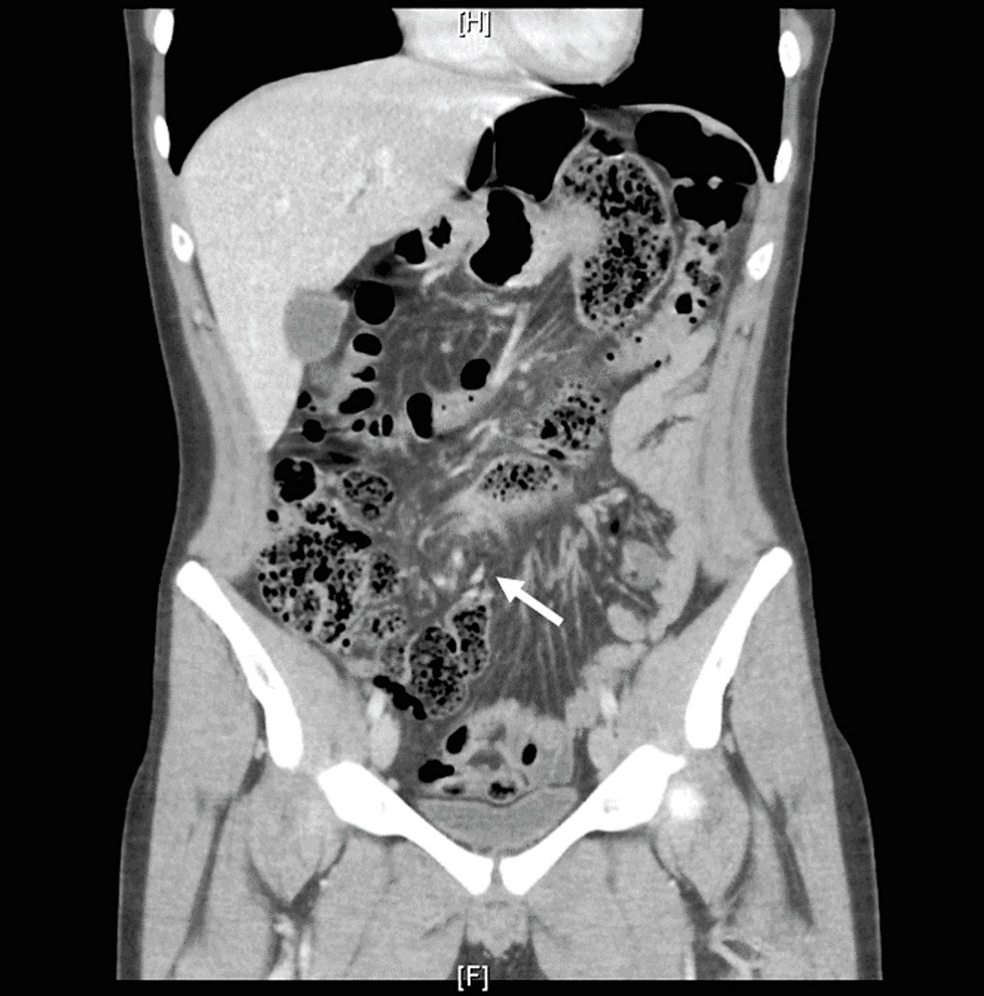
Coronal view CT of the abdomen and pelvis The white arrow is directed toward the swirling mesentery.

**Figure 2:**
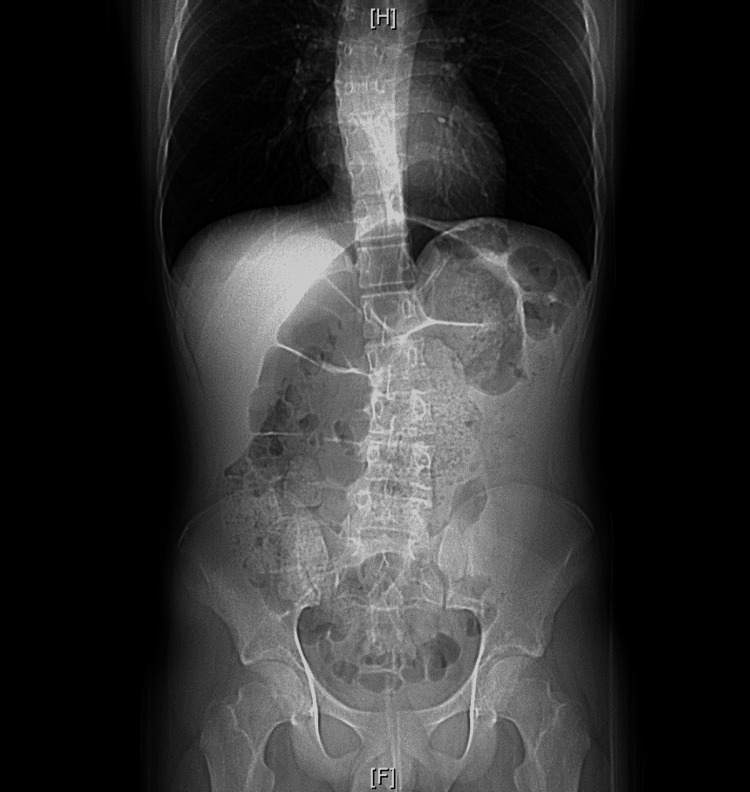
Scout view of the abdomen and pelvis There is a large dilated loop of the colon suggesting bowel obstruction and sigmoid volvulus.

**Figure 3:**
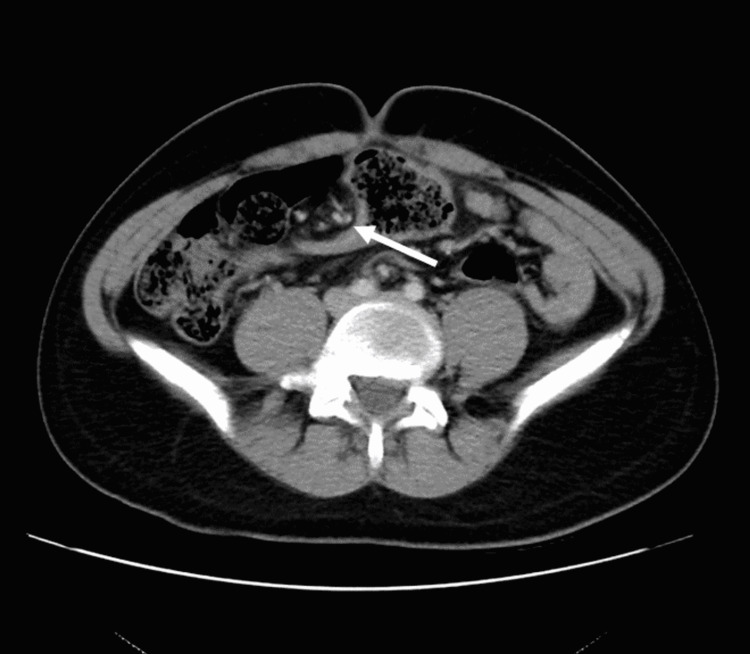
Transverse view CT of the abdomen and pelvis The white arrow is pointing to the “swirl sign” suggestive of sigmoid volvulus.

On presentation, he was diaphoretic-appearing with vital signs within normal limits. His physical examination was notable for suprapubic tenderness to palpation without distention, rigidity, guarding, or rebound tenderness. Additionally, McBurney’s point tenderness and Rovsing’s sign were negative, and the patient had no flank or costovertebral angle tenderness. The genitourinary examination was unremarkable. At the time of the examination, the differential included appendicitis, ureterolithiasis, constipation, and bowel obstruction. Notable laboratory results included a leukocytosis of 13.8 × 10^3^/µL (reference range: 4-10.5 × 10^3^/µL), a urinalysis without signs of infection or hematuria, and a negative chemistry panel. Due to a negative ultrasound (US) performed a week prior, the decision was made to proceed with CT, which subsequently showed swirling of the mesenteric vessels with a dilated proximal sigmoid colon consistent with sigmoid volvulus (Figures 1-3).

Intravenous fluids were started, and oral intake was changed to nil per os. Pediatric surgery was consulted, and the decision was made to transfer the patient to a tertiary facility with pediatric gastroenterology. Multiple attempts were made to contact the patient several weeks later, but he was subsequently lost to follow-up.

## Discussion

Sigmoid volvulus continues to remain a rare cause of abdominal pain in adolescent populations. A review of case reports from 1940 to 2000 identified only 63 cases of pediatric SV [10]. A more up-to-date review of the current literature suggests that the incidence may be increasing in younger individuals [3-5,9]. Theories have postulated that the cause is due to changes in Western diets and increasingly sedentary lifestyles. Risk factors in adolescents include a history of chronic constipation, a prior surgical history, Chagas disease, chronic debilitating conditions, and a history of Hirschsprung’s disease [3,6]. The pathology has a strong predilection for males regardless of age, although the onset of symptoms most commonly present in the seventh and eighth decades of life. Volvulus is uncommon in developed countries, with higher rates of incidence in countries that are a part of the “volvulus belt” such as those in the Middle East [11].

In the case of our patient, he presented with lower abdominal pain and one episode of emesis. He also noted a similar episode a week prior, which may or may not be related to his presenting complaint. His history illustrates two important potential presentations for the pathology. Sigmoid volvulus can present as acute fulminant volvulus with associated bowel obstruction or as a subacute presentation with partial obstruction and less severe symptoms [4]. Cases in the literature report intermittent sporadic detorsion as a possible presenting complaint [7,8]. Symptoms associated with complete obstruction are sudden in onset and include nausea and vomiting, while subacute presentations may be more insidious. Collectively, additional findings commonly include constipation, abdominal distention, and decreased bowel sounds.

Literature promoting the use of risk stratification tools and other evolutions in clinical practice, especially regarding the evaluation of abdominal pain in pediatric populations, has noticeably trended away from radiation-emitting modalities toward the use of US [12]. Despite increasing interest in US within the ED, the diagnostic sensitivity and specificity are limited by etiology, patient habitus, and operator ability [13]. Although additional research is needed, the accuracy of point-of-care ultrasound in bowel obstruction appears promising and may anecdotally have utility in the evaluation of SV [14]. X-rays are generally not reliable in diagnosis as classic signs such as a “coffee bean,” “omega,” inverted U or V signs are not present in one-third of adult patients [11]. In cases with a high index of suspicion, CT with contrast is the recommended modality with a sensitivity of approximately 100% and a specificity of greater than 90% [14]. Clinicians should avoid anchoring solely on the more common diagnosis of appendicitis and remain aware of the reliability and limitations of US. Anchoring bias may result in missing the even more debilitating, although rare, diagnosis of SV.

Definitive care is surgical with initial treatment being flexible sigmoidoscopy in cases where perforation is not suspected. Success rates with endoscopy in children are variable and reported to be greater than 47% [3,10]. Recurrence is common and often results in the removal of nonviable tissue with an end-to-end anastomosis or Hartmann procedure [6,15].

## Conclusions

Sigmoid volvulus is a rare cause of adolescent abdominal pain and should be considered in cases suggestive of intestinal obstruction. Clinicians should be careful not to anchor on the diagnosis and management of other more common “can’t miss” etiologies of abdominal pain such as appendicitis. Management often requires flexible sigmoidoscopy, with definitive treatment often resulting in elective resection to prevent recurrence.
